# Spatial lipidomics of coronary atherosclerotic plaque development in a familial hypercholesterolemia swine model

**DOI:** 10.1016/j.jlr.2024.100504

**Published:** 2024-01-19

**Authors:** Nuria Slijkhuis, Francesca Razzi, Suze-Anne Korteland, Bram Heijs, Kim van Gaalen, Dirk J. Duncker, Antonius F.W. van der Steen, Volkert van Steijn, Heleen M.M. van Beusekom, Gijs van Soest

**Affiliations:** 1 Department of Cardiology, Cardiovascular Institute, Thorax Center, Erasmus MC, Rotterdam, The Netherlands; 2 Department of Experimental Cardiology, Cardiovascular Institute, Thorax Center, Erasmus MC, Rotterdam, The Netherlands; 3 Department of Chemical Engineering, Delft University of Technology, Delft, The Netherlands; 4 Center for Proteomics and Metabolomics, Leiden University Medical Center, Leiden, The Netherlands; 5 Shenzhen Institutes of Advanced Technology, Chinese Academy of Sciences, Shenzhen, China; 6 Department of Imaging Science and Technology, Delft University of Technology, Delft, The Netherlands; 7 Department of Precision and Microsystems Engineering, Delft University of Technology, Delft, The Netherlands; 8 Wellman Center for Photomedicine, Massachusetts General Hospital, Boston, MA, USA

**Keywords:** Atherosclerosis, vascular biology, lipids/chemistry, dyslipidemias, inflammation, lipids, histology, mass spectrometry imaging, familial hypercholesterolemia, plaque progression

## Abstract

Coronary atherosclerosis is caused by plaque build-up, with lipids playing a pivotal role in its progression. However, lipid composition and distribution within coronary atherosclerosis remain unknown. This study aims to characterize lipids and investigate differences in lipid composition across disease stages to aid in the understanding of disease progression. Matrix-assisted laser desorption/ionization mass spectrometry imaging (MALDI-MSI) was used to visualize lipid distributions in coronary artery sections (n = 17) from hypercholesterolemic swine. We performed histology on consecutive sections to classify the artery segments and to investigate colocalization between lipids and histological regions of interest in advanced plaque, including necrotic core and inflammatory cells. Segments were classified as healthy (n = 6), mild (n = 6), and advanced disease (n = 5) artery segments. Multivariate data analysis was employed to find differences in lipid composition between the segment types, and the lipids' spatial distribution was investigated using non-negative matrix factorization (NMF). Through this process, MALDI-MSI detected 473 lipid-related features. NMF clustering described three components in positive ionization mode: triacylglycerides (TAG), phosphatidylcholines (PC), and cholesterol species. In negative ionization mode, two components were identified: one driven by phosphatidylinositol(PI)(38:4), and one driven by ceramide-phosphoethanolamine(36:1). Multivariate data analysis showed the association between advanced disease and specific lipid signatures like PC(O-40:5) and cholesterylester(CE)(18:2). Ether-linked phospholipids and LysoPC species were found to colocalize with necrotic core, and mostly CE, ceramide, and PI species colocalized with inflammatory cells. This study, therefore, uncovers distinct lipid signatures correlated with plaque development and their colocalization with necrotic core and inflammatory cells, enhancing our understanding of coronary atherosclerosis progression.

## Graphical Abstract

**Figure:**
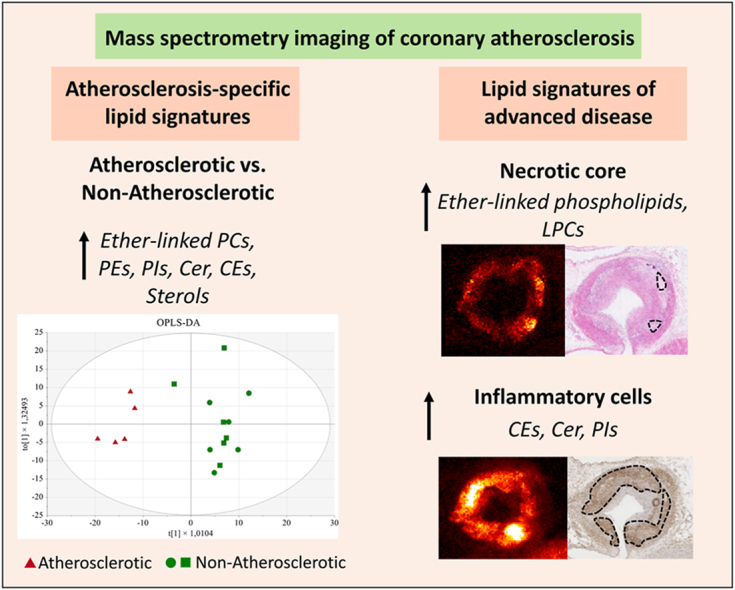

With around 18 million deaths annually, cardiovascular disease (CVD) is the leading cause of death worldwide (https://www.who.int/data/gho/data/themes/theme-details/GHO/mortality-and-global-health-estimates) (1). The main underlying pathology of CVD is atherosclerosis, which is characterized by the accumulation of lipids and inflammatory cells in the vessel wall (2, 3). Studying different disease stages of atherosclerosis can provide insights into disease pathogenesis, which can potentially lead to the identification of novel therapeutic targets for disease prevention or treatment. Given the crucial role of lipids in atherosclerosis development, understanding their specific contribution to disease initiation and progression is critical for advancing our understanding of disease pathogenesis.

A suitable technique for the detection of a wide range of lipids and the visualization of their distribution within plaque is matrix-assisted laser desorption/ionization mass spectrometry imaging (MALDI-MSI). MALDI-MSI is a molecular imaging technique capable of detecting and visualizing a wide range of lipids within tissue sections, operating in a label-free manner (4). This technique involves initially coating the tissue sections with an organic matrix, which aids in the crystallization of the analytes. The subsequent ionization of these analytes is achieved by directing a laser onto the matrix. The energy transferred from the laser to the matrix is pivotal in this ionization process, allowing for the precise detection of ions based on their mass-to-charge ratio in the mass detector.

Numerous studies have previously explored the spatial distribution and composition of lipids in carotid plaques using MSI. For instance, it was found that certain lipid species in carotid plaque colocalize with features of plaque instability, such as the colocalization of phospholipids with inflammation (5, 6, 7, 8). These studies have aided in enhancing our understanding of the lipidomic landscape in carotid atherosclerosis. However, there remains a need to extend this understanding to coronary atherosclerosis. Investigating lipid distribution in human coronary arteries is challenging, primarily because these samples are only available post-mortem. Consequently, the use of animal models serves as a valuable tool for the investigation of coronary atherosclerosis. Previous MSI studies have investigated the lipid distribution of coronary plaques in a mouse model (9, 10); however, these plaques often lack the physiological resemblance to human atherosclerosis.

A more suited animal model for the translation to human is the familial hypercholesterolemia (FH) swine model, as these swine develop plaques of varying sizes and compositions, ranging from healthy segments to complex atherosclerotic lesions that are similar to those observed in humans (11, 12). This is caused by a naturally occurring mutation in the low-density lipoprotein (LDL) receptor gene of FH swine leading to elevated LDL levels, as commonly observed in humans with a high risk of early onset atherosclerosis (13, 14).

In this study, we aimed to characterize the spatial distribution of lipids in coronary atherosclerosis in comparison to histology and, secondly, to investigate changes in lipid composition across various disease stages, from no plaque to progressive atherosclerotic lesions. For this purpose, we applied MALDI-MSI to visualize the lipid spectral patterns of coronary artery segments with varying disease stages in an adult FH swine model (12). In our analysis, we incorporate non-negative matrix factorization (NMF) for unsupervised clustering, identifying inherent patterns within the complex data. Additionally, we applied Principal Component Analysis (PCA) and Orthogonal Projections to Latent Structures Discriminant Analysis (OPLS-DA) to refine our data interpretation, focusing on simplifying data complexity and distinguishing between the lipid profiles of different disease stages, respectively. This approach, alongside a comparative analysis of lipid distributions and histology, aims to uncover lipid signatures in coronary atherosclerosis, revealing how these signatures vary with plaque development and their potential association with histological features of advanced disease.

## Materials and Methods

### Sample collection

All animal procedures were approved by the Animals Ethics Committee of the Erasmus University Medical Center Rotterdam, The Netherlands (EMC2125 (109-12-25)) and conducted in accordance with the National Institutes of Health Guide for Care and Use of Laboratory Animals and ARRIVE guidelines (15). For the present work, we analyzed coronary arteries procured from a previous study in which stent placement was investigated in arterial segments distant from the arterial segments used in this study (12). FH swine (n = 6 castrated male; French Bretoncelles-Meishan minipigs), homozygous for the LDLR R84C mutation as described before by Thim *et al.* (16), were used. Until the start of the study, the swine were provided with a standard laboratory diet (102,243/60, Sanders Ouest). Upon reaching an age of approximately 34±3 months, they were administered a high-fat diet (comprising 10% lard and 0.75% cholesterol, the National Institute of Agronomic Research) for 10 months to induce the development of atherosclerosis, as previously described (11, 12). After this period, at a mean age of 44±3, animals were euthanized and the heart was excised. From each animal, the three coronary arteries, left anterior descending (LAD), left circumflex (LCX), and right coronary artery (RCA), were carefully dissected free from the heart, preserving both the structural integrity of the artery itself and the surrounding periadventitial adipose tissue. Throughout the process, we ensured the preservation of a segment of the myocardium adjacent to the periadventitial adipose tissue for subsequent analysis. This procedure resulted in a total of 18 coronary arteries for histology and MALDI-MSI analysis. However, due to the failed dissection of one LCX artery from the heart, only 17 coronary arteries were processed further.

### Sample preparation for MALDI-MSI and histology

To perform MALDI-MSI measurements and histology, arteries were embedded in 10% porcine type A gelatin (Sigma-Aldrich) and 3 mm cross-sectional blocks of each artery were selected, snap-frozen, and kept at −80°C until cryosectioning. Cryosectioning (CM3050 S, Leica Biosystems) was performed at −20°C with a 10 μm cutting thickness. The sections were then thaw-mounted onto glass slides with two sections per slide and immediately stored at −80°C until further usage. For each artery segment, two glass slides were used for MALDI-MSI measurements in positive and negative ionization mode, and five sections for histology and immunohistochemistry. Additionally, two sections of the most diseased artery segment were used for high-mass resolution measurements using MALDI Fourier Transform Ion Cyclotron Resonance (FTICR) MSI in positive and negative ionization mode. Measurements were performed in both positive and negative ionization modes since different lipid classes may ionize more efficiently in one mode than the other, thus providing a broader coverage and deeper insight into the lipid composition of the sample. Before the MALDI-MSI measurements, sections were vacuum desiccated, and an organic matrix was deposited by sublimation (home-built sublimation system) (17). For positive ionization mode measurements, 2,5-dihydroxybenzoic acid (2,5-DHB) (Sigma Aldrich) matrix was used (50 mg dissolved in 5 ml acetone, sublimation for 10 min, 125°C), while for negative ionization mode measurements, 1,5-diaminonaphthalene (1,5-DAN) (Sigma Aldrich) matrix was used (50 mg dissolved in 5 ml acetonitrile, sublimation for 20 min, 145°C).

### MALDI-MSI measurements

MALDI-MSI measurements in positive and negative ionization mode were performed on a Synapt G2Si-TOF mass spectrometer with a MALDI source (Waters Corporation). The system was operated in resolution mode, utilizing a single-pass reflectron Time-of-Flight (TOF) with a mass resolution of 20,000. The MALDI source was equipped with a 1,000 Hz Nd:YAG (355 nm) laser with a pixel size of 45 × 45 μm^2^ which was controlled with Waters Research Enabled Software suite and fired with 100 shots per pixel. The mass range was *m/z* 300–1200. Data were acquired using MassLynx v4.2 software, and HDI v1.4 software (Waters Corporation) was used to export the data in imzML format. To process the data, we used a custom MATLAB™ 2017a (The Mathworks, Inc.) data-processing pipeline (18) in conjunction with mMass software (19) to select lipid *m/z* features and remove isotopes. For subsequent data analysis, we selected a subset of lipid *m/z* features that were present in at least 5 out of 17 artery sections.

### Lipid annotation

We assigned identities to lipid-related *m/z* features based on exact mass. Lipid *m/z* features were annotated by consulting the Lipid Maps database (20), wherein only annotations with a mass error under 15 parts per million were considered. To enhance the accuracy of the annotations, we conducted high-mass-resolution measurements using MALDI-FTICR-MSI on one of the five advanced diseased artery sections, in both positive and negative ionization mode. These measurements were performed using a Bruker Daltonics solariX xR mass spectrometer with a 12T superconductive magnet, a dynamically harmonized ParaCell™, and a Combi-Source™. The ftmsControl (v2.10 Build 98, Bruker Daltonics) was utilized to manage the system. Data acquisition involved a transient length of 3.3554 s (4M data points in the time domain), which resulted in an approximate resolution of 776,000 at *m/z* 400. The MALDI source was equipped with a SmartBeam™-II laser (355 nm) operating at 200 Hz and 15% power with the ‘’Small’’ focusing setting (ablation area approximately 70 × 70 μm^2^). The pixel size was 100 ×100 μm^2^ with 50 shots per pixel and a mass range of *m/z* 300–1200. Data were exported in imzML format using SCiLS Lab software (v2016b, Bruker Daltonics) for further processing. We processed the MALDI-FTICR-MSI data using a custom MATLAB™ 2017a (The Mathworks, Inc) data-processing pipeline (18) in combination with mMass software (19), utilizing a similar process as for MALDI-TOF-MSI data.

### Histology and tissue segmentation

The artery sections adjacent to the ones used for MALDI-MSI analysis were stained by hematoxylin & eosin (HE) as an overview stain (VWR), Oil Red O (ORO) as a lipid stain (Sigma Aldrich), resorcin-fuchsin (RF), as an elastin and collagen stain (Merck), and Anti-Cluster of Differentiation 68 (CD68) as immunohistochemical stain for monocyte/macrophages (cloneBA4D5, Abcam). Whole slides were digitized with a Nanozoomer 2.0HT slide scanner (Hamamatsu Photonics) at 20X magnification with a pixel size of 0.455 μm. The artery segments were classified based on histology by an experienced pathologist (HvB) according to disease severity as described before (11, 21) into the following three classes: no plaque (n = 6), non-atherosclerotic intimal lesions (n = 6; intimal thickening and intimal xanthoma), and progressive atherosclerotic lesions (n = 5; pathological intimal thickening and fibrous cap atheroma as evident from the presence of a necrotic core, which appeared as a lipid-rich area with few nuclei and fibrous tissue). For simplicity, we refer to these classes as healthy, mild-, and advanced disease artery segments, respectively.

Based on histology, tissue segmentation was performed in MeVisLab (MeVis Medical Solutions AG) annotating the following three segmentations: myocardium, periadventitial adipose tissue (PVAT), and artery (area bounded by the tunica adventitia). Within the artery segmentation of advanced disease, necrotic core and inflammatory cell regions were segmented. The segmentation images were registered to the MALDI tissue section by translation and scaling using an in-house developed point-based rigid image registration framework in MeVisLab, to enable correlation of histology and MALDI-MSI data. After registration, the mean and 99^th^ percentile max spectrum for each tissue segment were calculated per tissue section. To examine the colocalization of specific lipids with the histological tissue components necrotic core and inflammatory cells, two primary steps were employed. First, the 10% most intense pixels for each *m/z* feature were identified, representing the primary spectral pattern of the respective *m/z* images. Second, within each histological segmentation, the percentage of pixels overlapping with these top-intensity pixels was computed.

### Extracting the major spectral lipid patterns using unsupervised clustering

An unsupervised clustering algorithm, non-negative matrix factorization (NMF) (22), was applied to both the combined MALDI-MSI spectral data of all the entire tissue segmentations (including artery, PVAT, and myocardium), and solely on the artery segmentation across all tissue sections. This was done to identify the primary spectral lipid patterns and simplify the data’s complexity. An NMF toolbox for biological data mining was used (23). The number of optimal clusters was determined using a K-means algorithm.

### Lipid characterization of different segments using multivariate data analysis

Multivariate data analysis was performed in SIMCA 17 (Umetrics) to find differences in the lipid composition between different tissue segmentations. The variables used for the models were the 99^th^ percentile max intensities of all lipid-related *m/z* features from positive- and negative ionization modes combined. For the investigation of the differences in lipid composition between myocardium (n = 17), PVAT (n = 17), and artery (n = 17), Principal Component Analysis (PCA) and Orthogonal Projections to Latent Structures Discriminant Analysis (OPLS-DA) were employed with as observations the myocardium, PVAT, and artery segmentations.

PCA and OPLS-DA were also performed for the investigation of the differences in lipid composition between healthy artery segments (n = 6), artery segments with mild disease (n = 6), and artery segments with advanced disease (n = 5). For this analysis, the observations were all done on the artery segmentations. In the subsequent analysis, we combined healthy and mild artery segments to form a “non-atherosclerotic” group (n = 12) versus artery segments with advanced disease, now referred to as the “atherosclerotic” group (n = 5).

For the variables of these models, myocardium-specific *m/z* features were excluded based on the coefficients of the OPLS-DA discriminating myocardium from PVAT and artery, since these were not present in the artery segmentation.

For all OPLS-DA models, the quality of fit and predictability of the model were reported as *R*^2^ and Q^2^ values, respectively. The model was further validated by sevenfold cross-validation analysis of variance (CV-ANOVA) and permutation testing. The Variable Influence on Projection (VIP) values were extracted and VIP>1.0 were considered to have a significant influence on the separation in the model. Additionally, coefficients, which represent the specific influence of each variable on distinguishing between the predefined classes, were analyzed to gain deeper insights into their contributions to the class separation.

## Results

### MALDI-MSI visualized 473 lipid-related *m/z* features from 18 different lipid classes

**Fig. 1:**
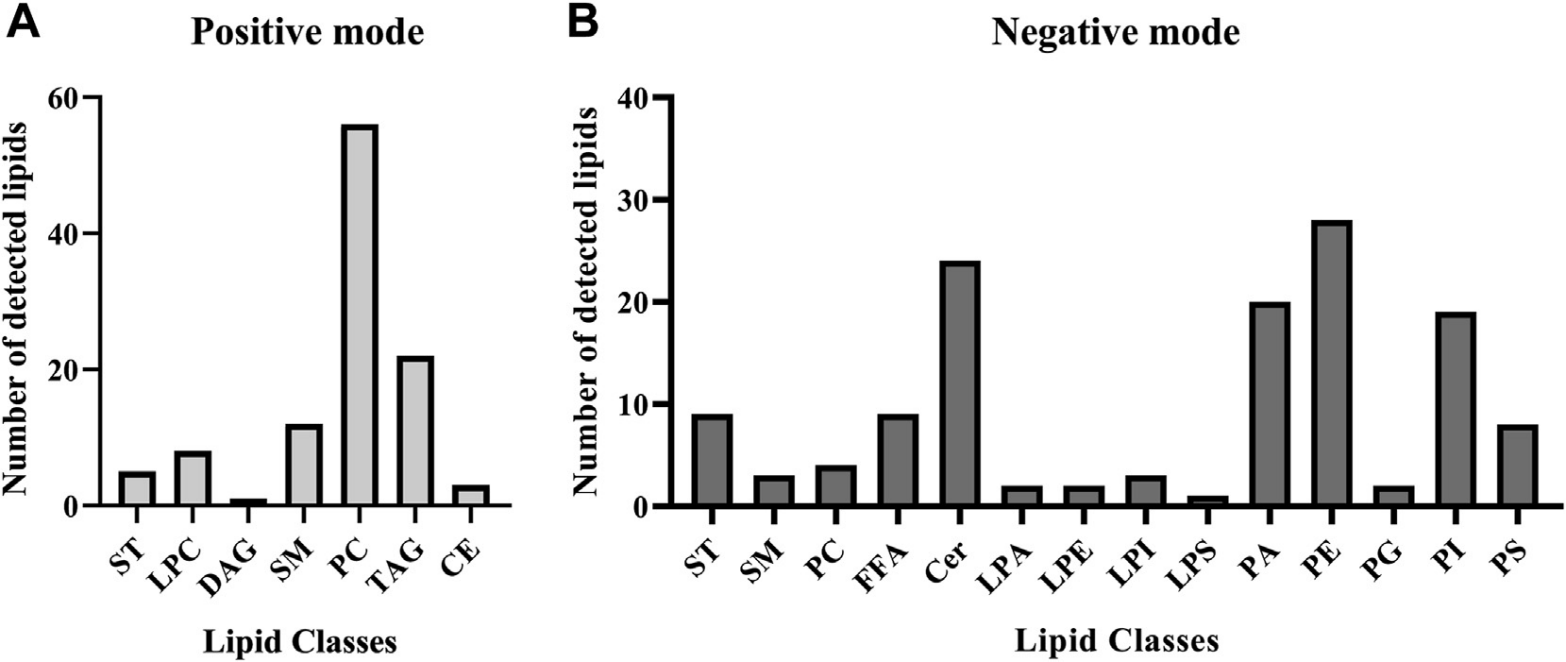
Number of detected lipids per lipid class by MALDI-MSI. A: Number of annotated lipid-related *m/z* features per lipid class in positive ionization mode. B: Number of detected lipids per lipid class in negative ionization mode. Cer species include CerP, CerPI, CerPE, HexCer, and Hex2Cer species.

MALDI-MSI visualized 473 lipid-related *m/z* features in the tissue sections (artery, PVAT, and myocardium), 235 in positive ionization mode, and 238 in negative ionization mode. Of these 473 *m/z* features, 241 could be annotated, see supplemental Tables S1 and S2 for the list of all *m/z* with lipid annotations in positive ionization mode and negative ionization mode, respectively. Lipids belong to 18 different lipid classes. In positive ionization mode, these lipid classes were: sterols (ST, including cholesterol), lysophosphatidylcholine (LPC), phosphatidylcholines (PC), sphingomyelins (SM), cholesteryl esters (CE), diacylglycerols (DAG), and triacylglycerols (TAG). In negative ionization mode, we detected the following lipid classes: free fatty acids (FFA), ST, lysophosphatidic acid (LPA), lysophosphatidylethanolamine (LPE), lysophosphatidylserine (LPS), lysophosphatidylinositol (LPI), phosphatidic acids (PA), phosphatidylethanolamine (PE), phosphatidylserine (PS), phosphatidylinositol (PI), and different classes of ceramides (Cer) including ceramide phosphoethanolamines (CerPE), ceramide phosphoinositiols (CerPI), ceramide 1-phosphates (CerP), hexosylceramide (HexCer), and lactosylceramide (Hex2Cer). The number of annotated lipid-related *m/z* features per lipid class in positive and negative mode is presented in Figure 1A, B, respectively.

### Unsupervised clustering revealed the major spectral lipid patterns

Initially, NMF clustering was applied to entire tissue sections, including the artery, myocardium, and PVAT, see supplemental Fig. S1 (positive; 3 components) and S2 (negative mode; 2 components) for the NMF spectra and corresponding NMF-weighted images, and supplemental Table S3 (positive) and S4 (negative mode) for the list of *m/z* features driving the NMF components. The NMF components primarily corresponded to the major tissue types of artery, myocardium, and PVAT (with some overlap). In positive mode, component 1 was associated with the myocardium and prominently influenced by PC species. Component 2, shared between the artery and PVAT, was driven by a different set of PC species, with a smaller contribution from SM species. Component 3, in the artery and myocardium, was driven by TAG species and cholesterol. In negative mode, component 1 was associated with artery and PVAT and was driven by Cer species, different phospholipid species, such as PE, PA, and PI, and by ST species. Component 2 was linked to the myocardium and was driven by a mix of phospholipids as well, with PI(38:4) having the most influence.

**Fig. 2:**
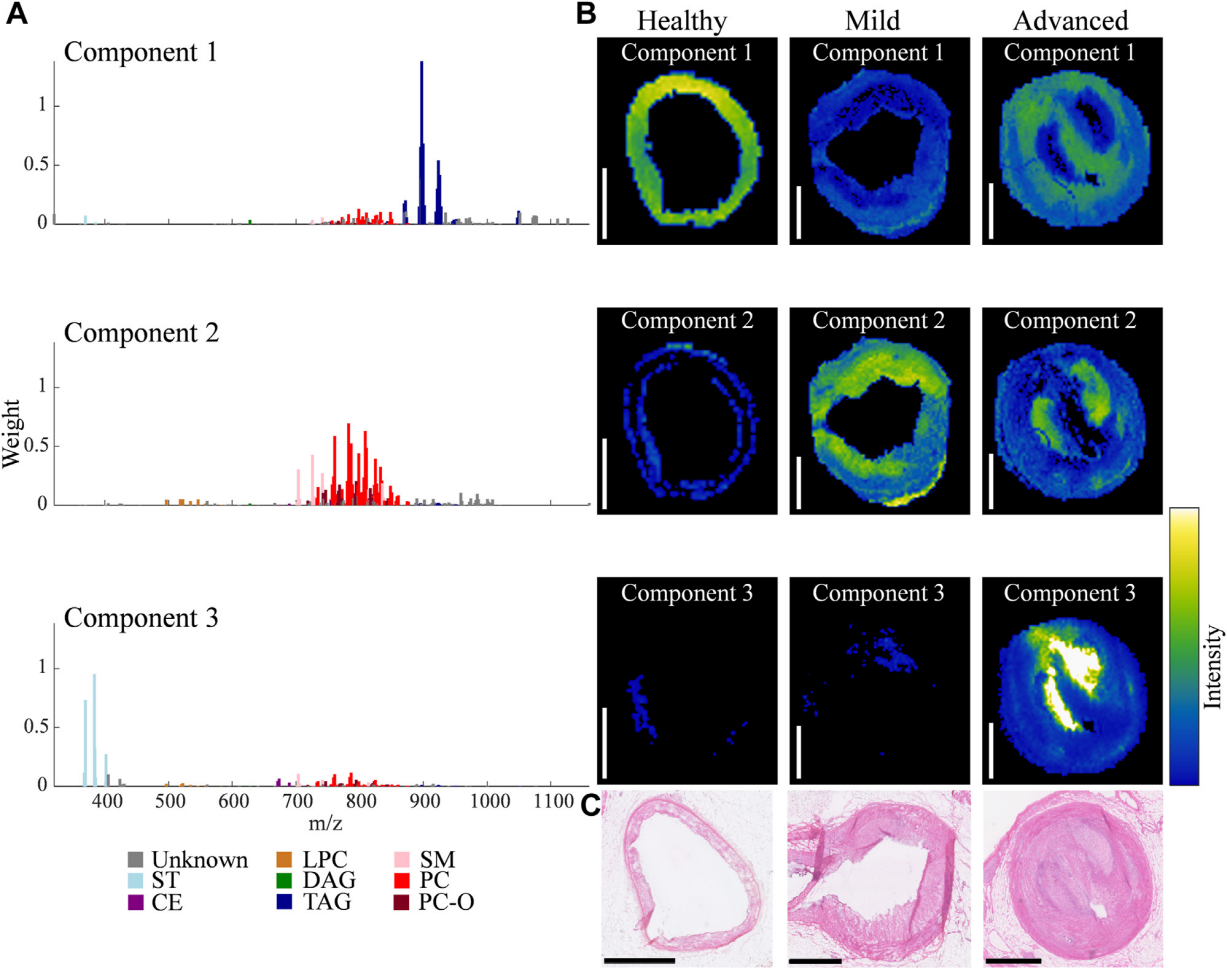
Unsupervised non-negative matrix factorization (NMF) of the 235 lipid-related *m/z* features in artery segments detected by MALDI-MSI in positive ionization mode. A: NMF spectra of the components showing the weight of each *m/z* feature relative to the corresponding component, *m/z* features are labeled based on their assigned lipid class. B: Corresponding NMF-weighted images of one representative section for each class (healthy, mild, and advanced disease artery segments), showing the spatial distributions and relative intensities of the NMF components. C: Corresponding H&E staining for the depicted artery segments. Sections from the following arteries were used: right coronary artery (healthy and mild) and left anterior descending artery (advanced). Scalebars = 1 mm.

Application of NMF to the artery segmentations only showed the spectral patterns in the arteries, and plaque in particular, in more detail. NMF clustering of the artery segmentations described the positive ionization mode data in three components, see Figure 2 for the NMF spectra with corresponding NMF-weighted images. Component 1 was driven by TAG species, component 2 was driven by PC species, and component 3 was mainly driven by cholesterol (and its derivatives) and 7-ketocholesterol, and by a combination of certain SM, CE, and PC species, see supplemental Table S5. The comparison of the distributions of the three components with histology revealed that component 1 was mostly present in the tunica media of both healthy artery segments and artery segments with advanced disease. Component 2 was mainly located throughout the whole plaque area and in healthy intima and adventitia, while component 3 was more pronounced in specific plaque areas, mostly present in the advanced-diseased artery segment.

**Fig. 3:**
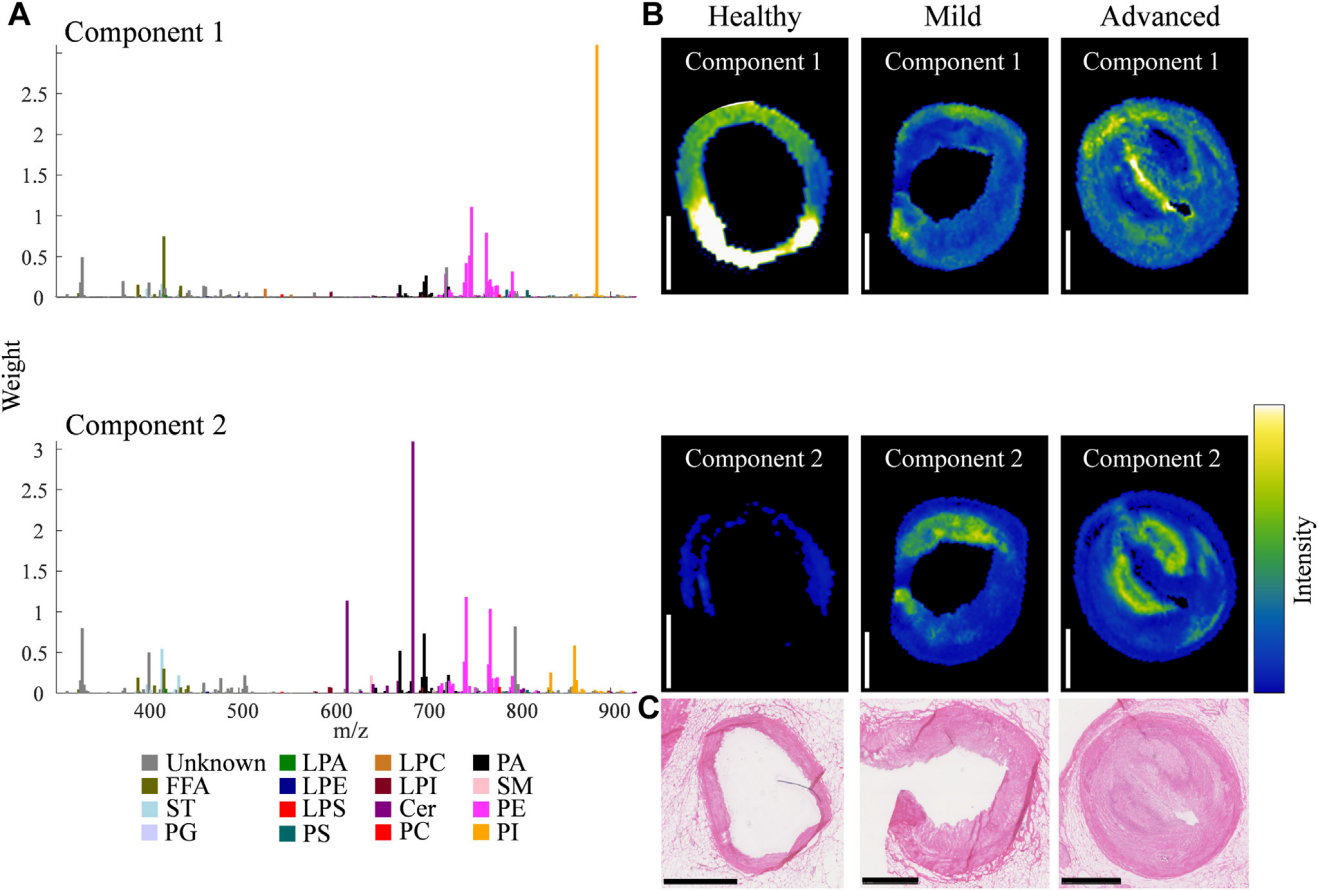
Unsupervised non-negative matrix factorization (NMF) of the 238 lipid-related *m/z* features detected in artery segments by MALDI-MSI in negative ionization mode. A: NMF spectra of the components showing the weight of each *m/z* feature relative to the corresponding component, *m/z* features are labeled based on their assigned lipid class. B: Corresponding NMF-weighted images of one representative section for each class (healthy, mild, and advanced disease artery segments), showing the spatial distributions and relative intensities of the NMF components. C: Corresponding H&E staining for the depicted artery segments. Sections from the following arteries were used: right coronary artery (healthy and mild) and left anterior descending artery (advanced). Scalebars = 1 mm.

The negative ionization mode data could be described by two components, see Fig. 3 for the NMF spectra with corresponding NMF-weighted images. Component 1 was mainly driven by PI(38:4), and to a lesser extent by a mix of PE, PA, and FFA species. Component 2 was driven by CerPE(36:1) primarily, with a prominent influence of CerP(34:1), and a mix of different phospholipids as well, see supplemental Table S6. The comparison of the distributions of the two components with histology revealed that component 1 was located in healthy media, in specific regions in the outer border of mild and advanced disease, and in the fibrous cap of advanced disease. Component 2 was located in the more diseased parts of the tissue, exemplified by the advanced disease shown in Figure 3B, where the distribution of component 2 is associated with the necrotic core.

### Multivariate data analysis highlights tissue-specific lipid signatures

We performed multivariate data analysis to find differences in the lipid composition between different tissue segmentations (myocardium, PVAT, and artery). PCA demonstrated clear clustering of the myocardium samples, while the PVAT and a part of the artery samples formed a cluster together, these were mainly healthy artery segments and mild disease artery segments. Artery segments with advanced disease formed a distinct cluster, indicating specific lipid signatures associated with advanced disease, see supplemental Fig. S3 for the PCA score plot.

Next, we applied OPLS-DA to discriminate between the myocardium, PVAT, and artery. The OPLS-DA model showed a clear separation between the segmentations, see supplemental Fig. S4. The model yielded an *R*^2^ value of 0.87 and a Q^2^ value of 0.80 and was further validated by a *P*-value of 1.28e-29, as calculated by CV-ANOVA. The lipids with the highest influence on the discrimination between groups (*m/z* features with a high VIP value) were several different phospholipid species, ceramide species, free fatty acids, and sterols. See supplemental Table S7 for the complete list of *m/z* features with a high VIP value.

Subsequently, our analysis primarily focused on the artery segmentations while excluding specific *m/z* features, mainly phospholipids, that were exclusively associated with the myocardium and were absent in the artery segmentation based on the OPLS-DA model. We first fitted a PCA model with all artery segments, including advanced-diseased (n = 5), mild-diseased (n = 6), and healthy (n = 6). Artery segments with advanced disease formed a distinct cluster, while healthy and mildly diseased artery segments grouped, see supplemental Fig. S5.

We determined the lipids that were most discriminative between these two groups (healthy/mild disease vs. advanced disease) by computing an OPLS-DA model using a “non-atherosclerotic” (healthy or mild disease) group (n = 12) versus artery segments with advanced disease, now referred to as the “atherosclerotic” group (n = 5). The resulting OPLS-DA model demonstrated an *R*^2^ value of 0.88 and a Q^2^ value of 0.66 with a significant *P*-value of 0.0083. A three-group OPLS-DA model (healthy, mild disease, and advanced disease) did not yield statistically significant parameters.

**Fig. 4:**
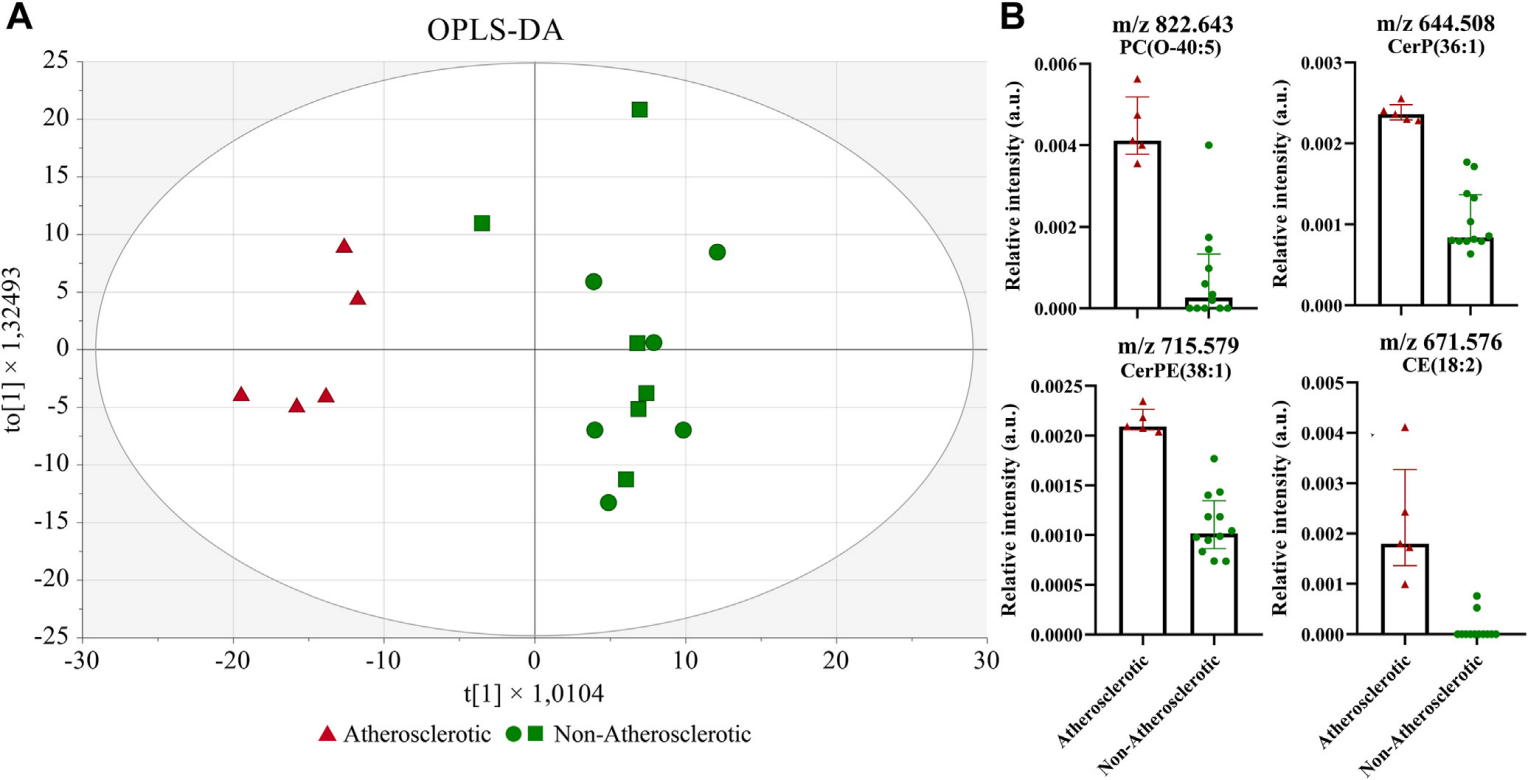
OPLS-DA score plot of the atherosclerotic vs. non-atherosclerotic artery segments and boxplots of *m/z* features with a high VIP value. A: OPLS-DA score plot. In the score plot, atherosclerotic artery segments are represented by a red triangle, while the non-atherosclerotic group is displayed in green, with the artery segments with a mild disease as green squares and the healthy artery segments as green circles. B: Boxplots of *m/z* features with a high VIP value, displayed are: PC(O-40:5) *m/z* 822.643, CerP(36:1) *m/z* 644.508, CerPE(38:1) *m/z* 715.579, and CE(18:2) *m/z* 671.576. The data are presented as median and interquartile range (IQR).

In the two-group model, the score plot showed a clear separation of the atherosclerotic and non-atherosclerotic artery segments, see Figure 4A. One non-atherosclerotic artery segment (with mild disease) was positioned between the atherosclerotic and non-atherosclerotic groups. Histological examination of this artery segment reveals that half of the artery segment displayed a healthy morphology, while the other half exhibited intimal thickening with lipids, as confirmed by ORO staining. Lipids with the highest influence on separating the groups were several ether-linked phosphatidylcholines, other phospholipids such as PE and PI species, Cer species, and CE and sterols. Figure 4B displays the boxplots of the relative intensity for individual lipid species with a high VIP in atherosclerotic and non-atherosclerotic samples, namely PC(O-40:5) *m/z* 822.643, CerP(36:1) *m/z* 644.508, CerPE(38:1) *m/z* 715.579, and CE(18:2) *m/z* 671.576, which all showed to be more pronounced in artery segments with advanced atherosclerosis. We further examined the coefficients to identify key lipid species associated with each class. For atherosclerotic artery segments, the most discriminating annotated *m/z* features were ST(28:1;O), PA(O-40:6), 7-ketocholesterol, ST(27:2;O), cholesterol, CE(18:2), LPI(18:0), CE(18:1), CerPE(38:1), and PC(O-40:5). For non-atherosclerotic artery segments, the coefficients generally remained lower, indicating a weaker influence of lipid species differentiating the non-atherosclerotic artery segments. For the complete list of VIP values and coefficients, see supplemental Tables S10 and S9.

### Spatial lipid patterns colocalize with histological features in advanced disease

**Fig. 5:**
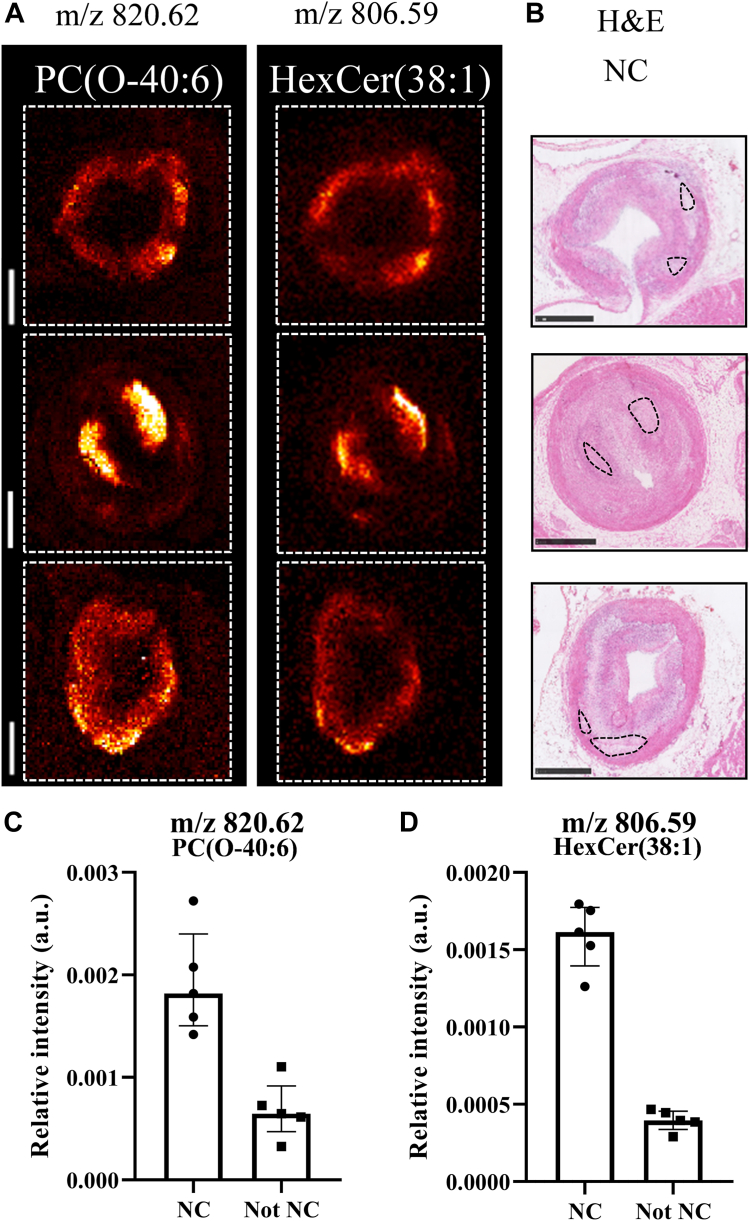
Colocalization of specific lipids and necrotic core. A: MALDI images of *m/z* 820.62 (PC(O-40:6)) and *m/z* 806.59 (HexCer(38:1)) of 3 different tissue sections. B: Corresponding H&E images with superimposed outlines of the necrotic core regions. C: Boxplots of *m/z* 820.62 and D: *m/z* 806.59 showing the mean intensity of the pixels in the necrotic core regions and in the non-necrotic core regions. The data are presented as median with IQR. Sections from the following arteries were used: left circumflex artery (top), left anterior descending artery (middle), and right coronary artery (bottom). Scale bars are 1 mm. NC, necrotic core.

In the positive ionization mode data, we observed colocalization of mostly ether-linked PC (50%–69% colocalization) and LPC (43%–58% colocalization) species with necrotic core, whereas in the negative ionization mode data, we found a colocalization of HexCer (75%) and ether-linked phospholipid species (PE O, PA O, and PI O) (45%–67%) with necrotic core. Figure 5A displays the spatial distributions of PC(O-40:6) and HexCer(38:1) alongside the H&E staining with annotated necrotic core regions (Fig. 5B). The mean intensity of the pixels of these selected lipids in necrotic core regions was higher compared to the intensity outside the necrotic core regions (Fig. 5C, D).

**Fig. 6:**
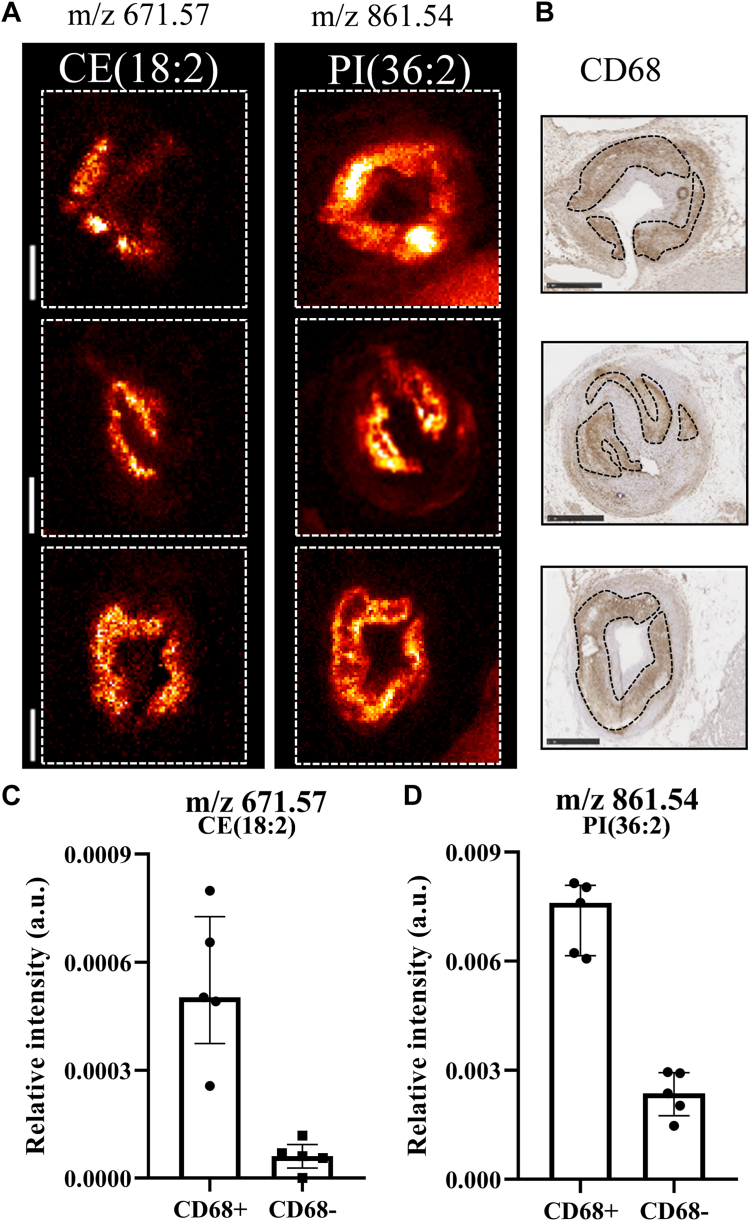
Colocalization of specific lipids and inflammatory cells. A: MALDI images of m/z 671.57 (CE(18:2)) and *m/z* 861.54 (PI(36:2)) of 3 different tissue sections. B: Corresponding CD68 stained images with superimposed outlines of the CD68-positive cell regions. C: Boxplots of *m/z* 671.57 and D: *m/z* 861.54 showing the mean intensity of the pixels in the CD68-positive cell regions and in the non-CD68-positive cell regions. The data are presented as median with IQR. Sections from the following arteries were used: left circumflex artery (top), left anterior descending artery (middle), and right coronary artery (bottom). Scale bars are 1 mm.

In the regions associated with inflammatory cells, we found a colocalization with CE (24%), SM (17%), and ST (20%) species in positive ionization mode. Additionally, in negative ionization mode, we identified a colocalization with Cer (23%–27%) and different phospholipids, mostly PI species (23%–27%). Figure 6 shows the spatial distribution of CE(18:2) and PI(36:2) alongside the CD68 staining with annotated CD68-positive cell regions. The mean intensity of the pixels of these selected lipids in CD68-positive cell regions was higher compared to the intensity outside (Fig. 5C, D). The complete list of lipids colocalizing with necrotic core and inflammatory cell regions can be found in supplemental Tables S10 and S11.

## Discussion

In this study, our primary objective was to examine the spatial distribution of lipids in coronary atherosclerosis in comparison to histology and, secondly, to investigate changes in lipid composition across various disease stages, from no plaque to progressive atherosclerotic lesions.

To achieve this, our study employs an FH swine model, known for its closer physiological resemblance to human atherosclerosis (11, 12). This model allowed us to characterize coronary plaques at various stages of atherosclerosis development effectively. In total, we imaged the spatial distribution of 473 lipid-related signals in 17 tissue sections obtained from swine coronary atherosclerotic plaques. By concurrently processing tissue sections adjacent to those used for MALDI-MSI for histology, we were able to validate and complement our lipid-related findings with traditional histological assessments, thereby enhancing the overall reliability of our conclusions.

### Multivariate data analysis highlights plaque-specific lipid composition

The multivariate data analysis revealed insights into the lipid composition variations across different tissue segmentations, including myocardium, PVAT, and artery. The PCA results demonstrated clear and distinct clustering of the myocardium samples, while PVAT and a subset of artery samples formed a cluster together, primarily comprising artery segments belonging to the healthy or mild disease class, indicating similar lipid profiles. Conversely, artery segments with advanced disease formed a distinct cluster separate from PVAT and healthy or mildly diseased artery segments. Functionally, PVAT, with its dynamic metabolic environment, actively participates in vascular homeostasis. Its direct contact with the vascular wall enables PVAT to exert paracrine effects on adjacent vascular tissues, with the potential to impact vascular tone, inflammation, and other critical processes through the secretion of bioactive molecules (24). While the lipid profiles of PVAT and the artery wall may mirror each other in healthy or mild disease states, our findings suggest that this similarity does not extend to late-stage vascular pathology.

To further investigate which lipids characterize coronary atherosclerosis, we focused on the artery segmentations only for the subsequent analysis. Artery segments were classified based on histological examination into the following classes: healthy artery segments, artery segments with mild disease, and artery segments with advanced disease. PCA of these three classes, revealed two clusters, one with artery segments with advanced disease and one with both healthy artery segments and artery segments with mild disease together. This observation suggests a potential similarity in lipid profiles between mildly diseased and healthy artery segments. This similarity was further confirmed by an OPLS-DA model, which was not significant and showed no lipids specifically associated with artery segments with mild disease. These findings underscore the transitional nature of the mild disease stage, potentially representing an intermediate state between healthy vessels and advanced disease. The absence of specific lipid associations in mild disease artery segments indicates that it may not be lipid-driven. This finding is in line with the hypothesis that most atherosclerotic lesions originate from preexisting intimal cell masses. These intimal cell masses refer to accumulations or clusters of cells in the intima layer of the artery, which can grow and evolve before lipid accumulation occurs leading to the development of atherosclerotic lesions (21, 25).

In line with the PCA results, we grouped healthy artery segments and artery segments with mild disease together as a “non-atherosclerotic” group compared to the advanced samples labeled as the “atherosclerotic” group, which yielded a significant OPLS-DA model discriminating both groups. The lipids that primarily differentiated the atherosclerotic group belonged to the lipid classes ST, ether-linked phospholipids, CE, and Cer. To gain a more detailed understanding of the location of the lipids resulting from NMF and OPLS-DA analysis, we compared lipid distributions to histological regions of interest by segmenting the necrotic core and regions rich in inflammatory cells.

### Spatial lipid patterns colocalize with histological features of artery segments with advanced disease

The necrotic core colocalized mostly with ether-linked PCs, Ceramides, PCs, and lysophospholipids. LPCs and ether-linked species, in particular LPC(18:1) and PC(O-34:1) are known to be correlated with cardiovascular diseases (26). Furthermore, a connection between ether-linked phospholipids and ferroptosis, a non-apoptotic programmed cell death pathway, has been proposed (27, 28), which could explain their colocalization with necrotic core. Furthermore, all ether-linked phospholipids colocalizing with necrotic core were unsaturated which makes them prone to oxidation, thereby producing pro-inflammatory substances, such as LPC (29). Previous studies have established a correlation between LPC and plaque instability, as LPC can induce the expression of cell adhesion molecules in endothelial cells (30). This class of lipids comprises some of the key bioactive molecules found in oxidized low-density lipoprotein, and it plays a significant role in various conditions linked to vascular oxidative stress and inflammation (31, 32). Notably, elevated levels of oxidative stress within cells can cause substantial harm to cellular components, often resulting in necrosis (33).

Certain species of SM, a lipid class that has been associated with plaque formation (9, 34, 35), were additionally observed to colocalize with necrotic core. SM is a type of sphingolipid, a class of lipids found in cell membranes. They play various roles in cell signaling, membrane structure, and cell function (36). In the context of atherosclerosis, high levels of SM in lipoproteins have been suggested to promote atherogenesis (37, 38). Furthermore, SM is converted by acid sphingomyelinase enzyme activity into Cer, which can stimulate cell death and inflammatory responses which are critical in the development of atherosclerosis (39). The observation of SM presence in necrotic core is consistent with previous research conducted on carotid plaques (5). Our study revealed that the elevated levels of the discussed lipids are specifically concentrated in the necrotic cell regions of the plaque.

CEs and phospholipids were primarily found to colocalize with CD68-positive cell areas. Previous studies have also reported the occurrence of CE and PC species in plaques, with their levels increasing as lesion complexity grows, thus establishing a connection between these lipids and cardiovascular diseases (26). Furthermore, we observed a robust colocalization of CE(18:1) and CE(18:2) with CD68-positive cell areas. While colocalization between lipids and inflammatory cells was confirmed, the lower colocalization percentages observed can be attributed to the smaller structural size of inflammatory cells relative to the lipid spectral patterns, suggesting that the lipid signals may span larger areas than the compact regions occupied by the cells. The literature extensively documents the close relationship between plasma lipids, vascular matrix, and CE deposits in the arterial wall (40, 41). During atherosclerosis development, macrophages transform into foam cells as they accumulate CE droplets (41, 42, 43). PI species were also found to colocalize with CD68-positive cell areas. PI is a type of phospholipid that plays a critical role in cell signaling and membrane function (44). Focused studies on specific PI species and their direct impact on atherosclerosis are still limited. However, it is worth noting that phospholipids, including PIs, are essential components of cell membranes and are involved in various cellular processes, including inflammation and immune response. Changes in the composition of phospholipids, including PI, may influence cellular functions and contribute to the development or progression of atherosclerosis. Furthermore, we identified the NMF component driven by PI(38:4) as being notably present in the myocardium, rich in cardiomyocytes, and within the fibrous cap of arteries, which is abundant in smooth muscle cells. Both regions inherently contain muscle cells, hinting at shared functional relevance. Given the presence of macrophages in the fibrous cap as well, PI(38:4) might be involved in modulating the activities of muscle cells and/or macrophages during atherosclerosis.

### Study limitations

While human samples would ideally provide the most direct insights into coronary atherosclerosis, our study utilized the FH swine model due to its similarity to human disease pathology. The small sample size, inherent to this model, may restrict the generalizability of our findings. Despite this, our findings enhance our understanding of the lipidomic dynamics in coronary atherosclerosis and could guide future studies with larger sample sizes or direct human samples. Furthermore, while histology with ORO staining offered valuable insights into the composition of neutral lipid plaques, it was unable to detect other polar lipids present in the tissue sections. To bridge this gap and complement the MALDI-MSI data, there is a pressing need to develop new histology staining techniques capable of detecting polar lipids. Additionally, the artery segments utilized for MALDI-MSI might not accurately represent the entire state of the artery from which they were sourced. Finally, histological examination of the arterial tissue allowed us to categorize six artery segments as healthy. However, it is important to consider that these artery segments were obtained from an animal model with induced disease, which could potentially influence the appearance of healthy intima.

## Conclusion

Our study provided novel insights into the lipid distributions in arterial health and disease, differentiating developmental stages of atherosclerosis. We found that the lipid profile of mildly diseased artery segments resembled healthy tissue, suggesting this disease state is not lipid-driven. There was a distinct lipid signature associated with advanced disease. Additionally, our analysis revealed the colocalization of these plaque-related lipids with necrosis and inflammation within the arterial tissue. These findings can contribute to a better understanding of disease pathogenesis.

## Data Availability

Data can be shared upon request. Please contact Prof. Gijs van Soest, Erasmus MC, g.vansoest@erasmusmc.nl.

## Supplemental data

This article contains supplemental data.

## Conflict of interest

The authors declare the following financial interests/personal relationships which may be considered as potential competing interests:

Gijs van Soest is an advisor to, and has a financial interest in, Kaminari Medical BV. The other authors have no conflicts of interest to declare.
