# Supplementary material for: Spatial lipidomics of coronary atherosclerotic plaque development in a familial hypercholesterolemia swine model
Source: J Lipid Res. 2024 Jan 19;65(2):100504. doi: 10.1016/j.jlr.2024.100504 (PMC10879031; doi:10.1016/j.jlr.2024.100504)
Supplement: Supplemental Material [file mmc1.docx]

**Supplemental Material**

**Spatial Lipidomics of Coronary Atherosclerotic Plaque Development in a Familial Hypercholesterolemia Swine Model**

*Nuria Slijkhuis*, Francesca Razzi*, Suze-Anne Korteland, Bram Heijs, Kim van Gaalen, Dirk J. Duncker, Antonius F.W. van der Steen, Volkert van Steijn, Heleen M. M. van Beusekom†, Gijs van Soest†*

**Please refer to the .xlsx supplemental file for the following tables:**

**Table S1:** List of *m/z* with annotations measured in the artery sections in positive ionization mode. ^a^ = Lipid maps; ^b^ = High mass resolution FTICR; ^c^ = Literature. Blanks are unknown.

**Table S2:** List of *m/z* measured in the coronary arteries in negative ionization mode. ^a^ = Lipid maps; ^b^ = High mass resolution FTICR; ^c^ = Literature. Blanks are unknown.

**Table S3**: NMF whole tissue sections – List of m/z features per NMF component positive ion mode

**Table S4**: NMF whole tissue sections – List of m/z features per NMF component negative ion mode

**Table S5:** NMF artery segments only - List of *m/z* features per NMF component positive ion mode

**Table S6:** NMF artery segments only - List of *m/z* features per NMF component in negative ion mode

**Table S7:** VIPs OPLS-DA myocardium – periadventitial fat - artery

**Table S8:** VIPs OPLS-DA model Atherosclerotic vs non-atherosclerotic

**Table S9:** Coefficients from the OPLS-DA model atherosclerotic vs non-atherosclerotic group

**Table S10:** Lipids co-localizing with necrotic core in positive and in negative ionization mode

**Table S11:** Lipids co-localizing with inflammatory cells in positive and in negative ionization mode

**
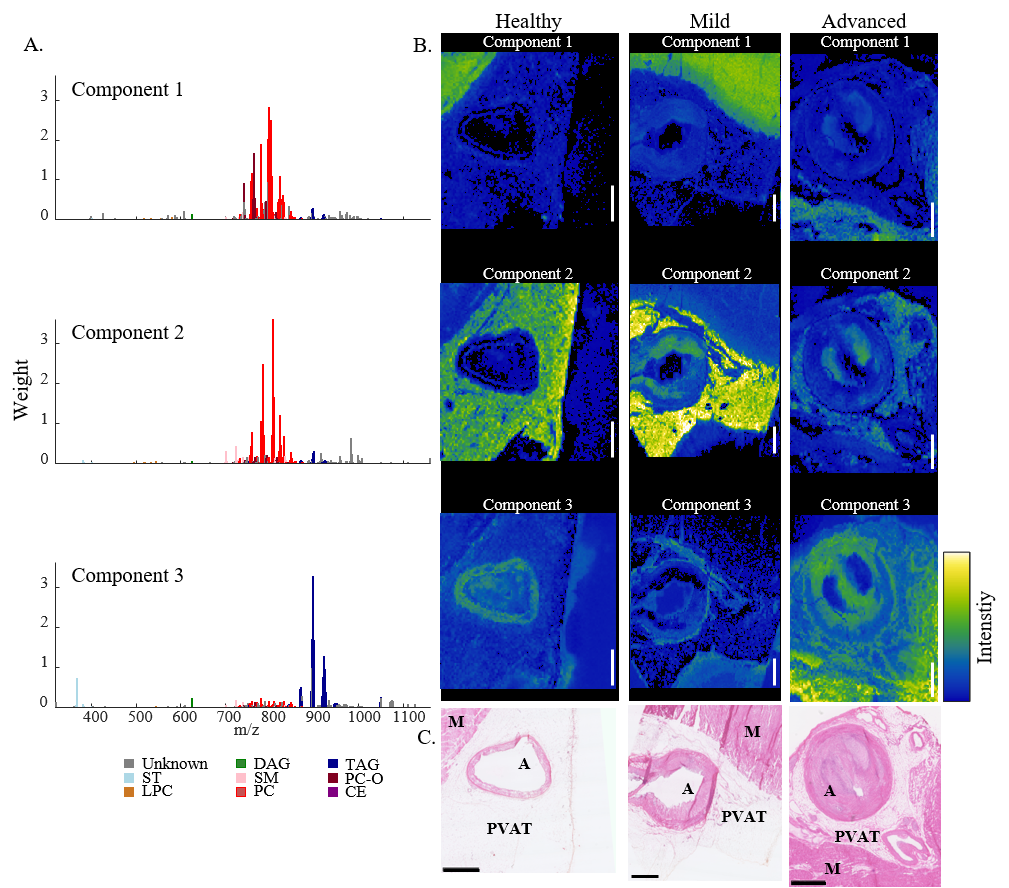
Figure S1: Unsupervised non-negative matrix factorization (NMF) of the 236 lipid-related *m/z* features in the whole tissue section (including the artery, myocardium, and periadventitial adipose tissue) detected by MALDI-MSI in positive ionization mode.** A) NMF spectra of the components showing the weight of each *m/z* feature relative to the corresponding component, *m/z* features are labelled based on their assigned lipid class. B) Corresponding NMF-weighted images of one representative section for each class (healthy artery, mild and advanced disease arteries), showing the spatial distributions and relative intensities of the NMF components. C) Corresponding H&E staining for the depicted arteries. H&E images are reused from Figure 2C in the manuscript. Sections from the following arteries were used: right coronary artery (healthy and mild) and left anterior descending artery (advanced). A = artery; M = myocardium; PVAT = periadventitial adipose tissue. Scale bars are 1 mm.

**
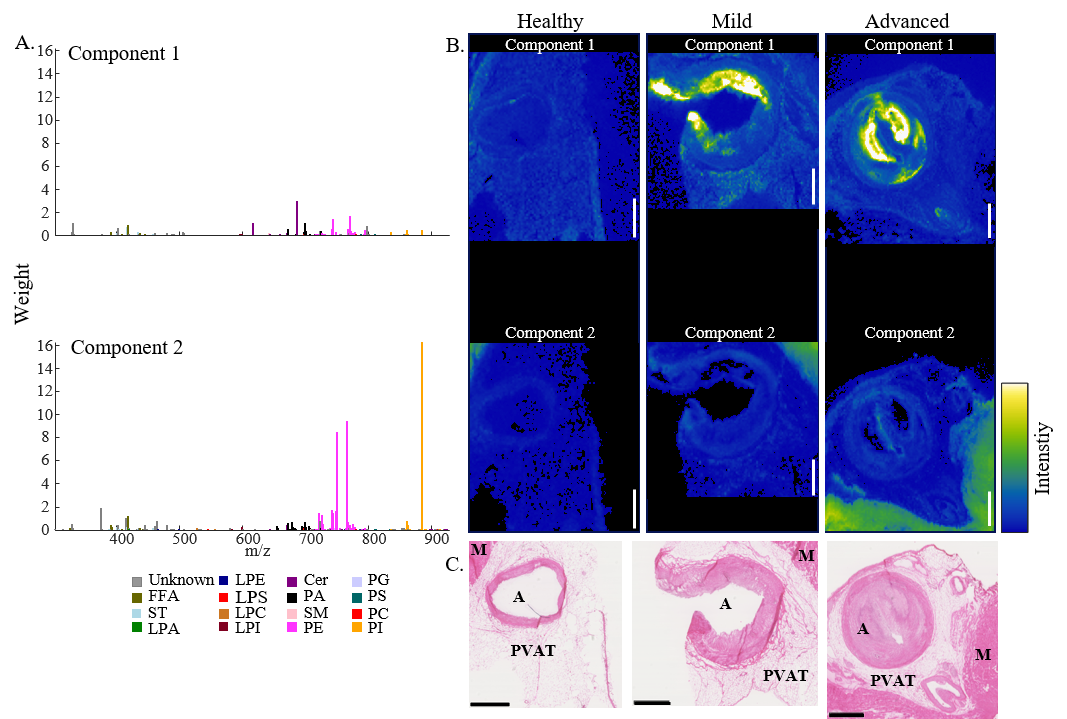
**

**Figure S2: Unsupervised non-negative matrix factorization (NMF) of the 237 lipid-related *m/z* features in the whole tissue section (including the artery, myocardium, and periadventitial adipose tissue) detected by MALDI-MSI in negative ionization mode.** A) NMF spectra of the components showing the weight of each *m/z* feature relative to the corresponding component, *m/z* features are labelled based on their assigned lipid class. B) Corresponding NMF-weighted images of one representative section for each class (healthy artery, mild and advanced disease arteries), showing the spatial distributions and relative intensities of the NMF components. C) Corresponding H&E staining for the depicted arteries. H&E images are reused from Figure 3C in the manuscript. Sections from the following arteries were used: right coronary artery (healthy and mild) and left anterior descending artery (advanced). A = artery; M = myocardium; PVAT = periadventitial adipose tissue. Scale bars are 1 mm.

**
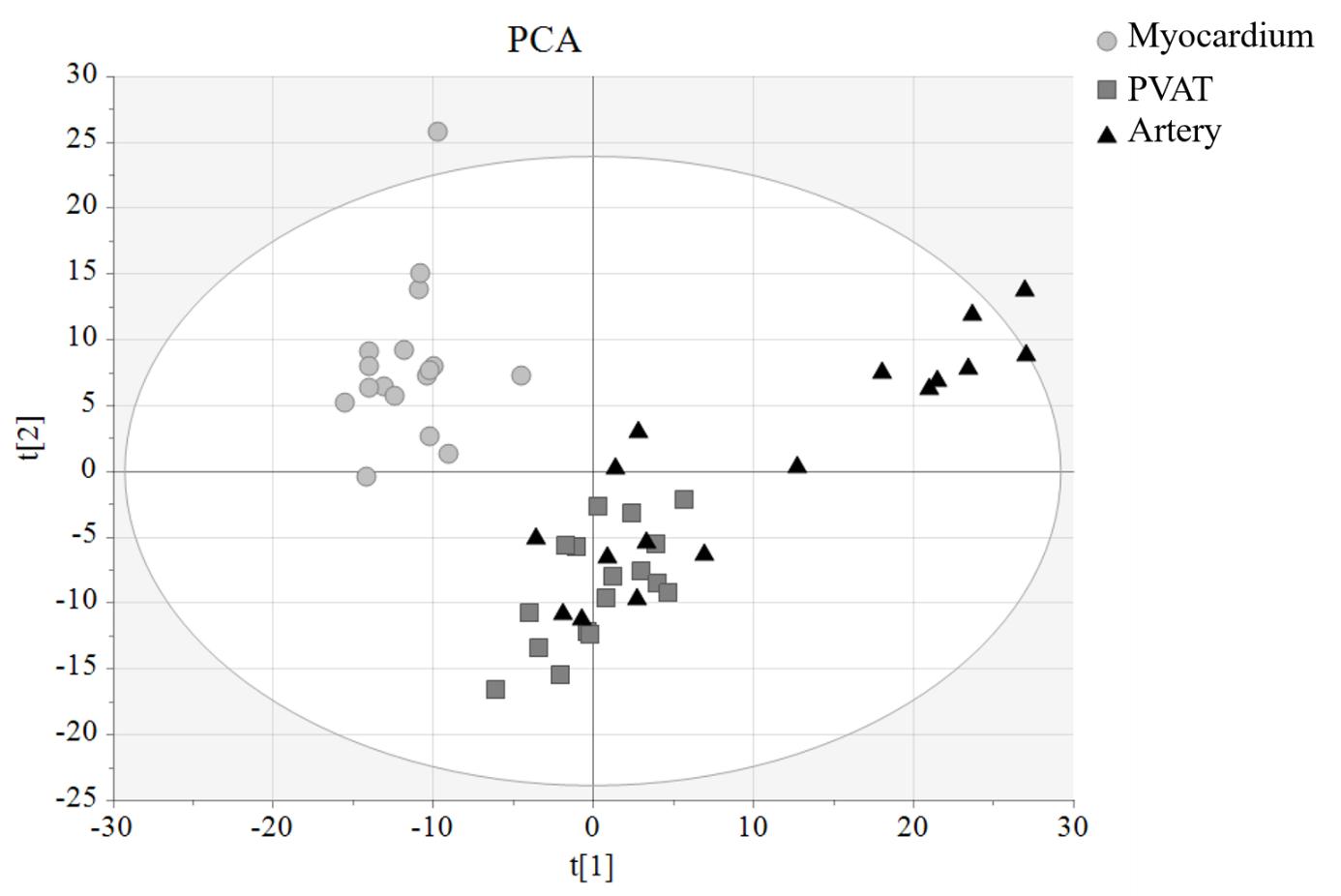
**

**Figure S3:** Score plot PCA analysis for segments myocardium, periadventitial adipose tissue (PVAT), and artery.


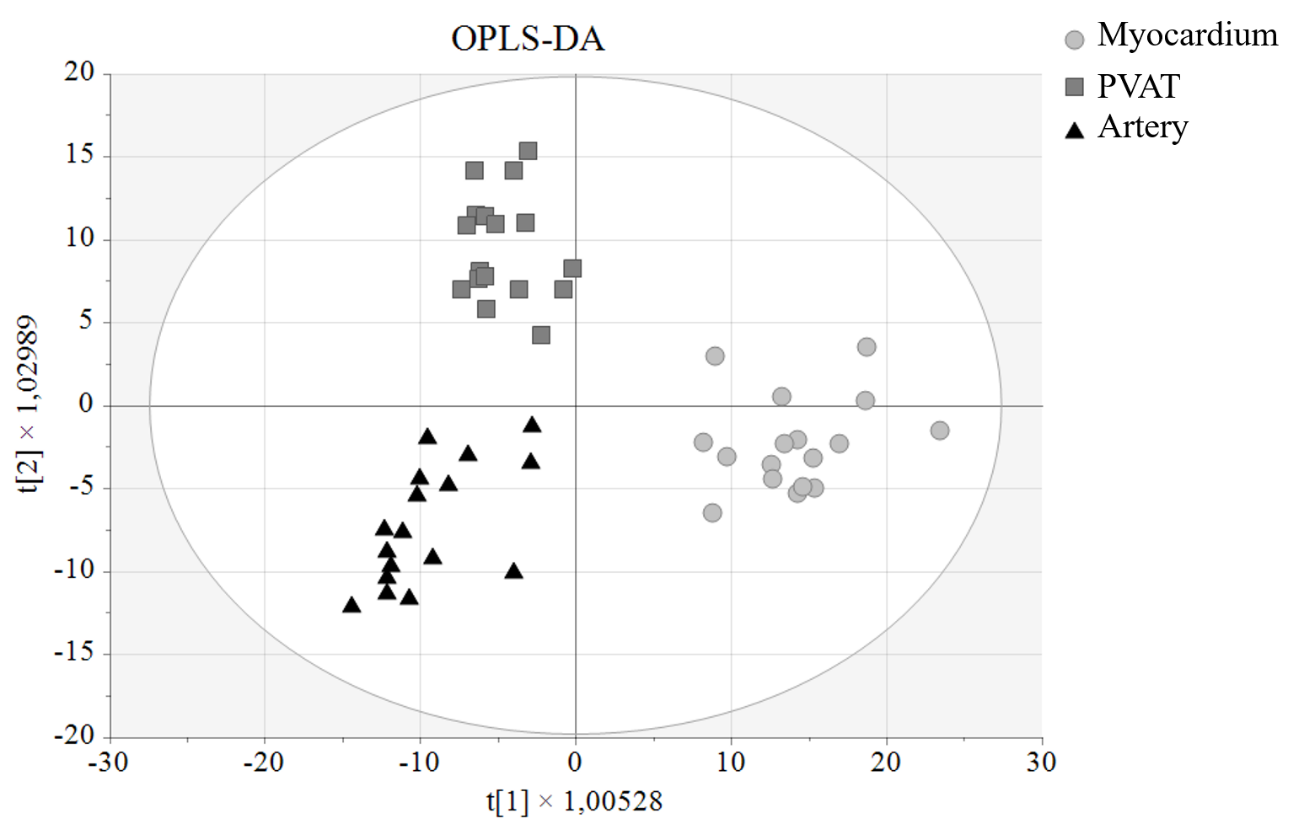
 **Figure S4:** Score plot OPLS-DA analysis for segments myocardium, periadventitial adipose tissue (PVAT), and artery.


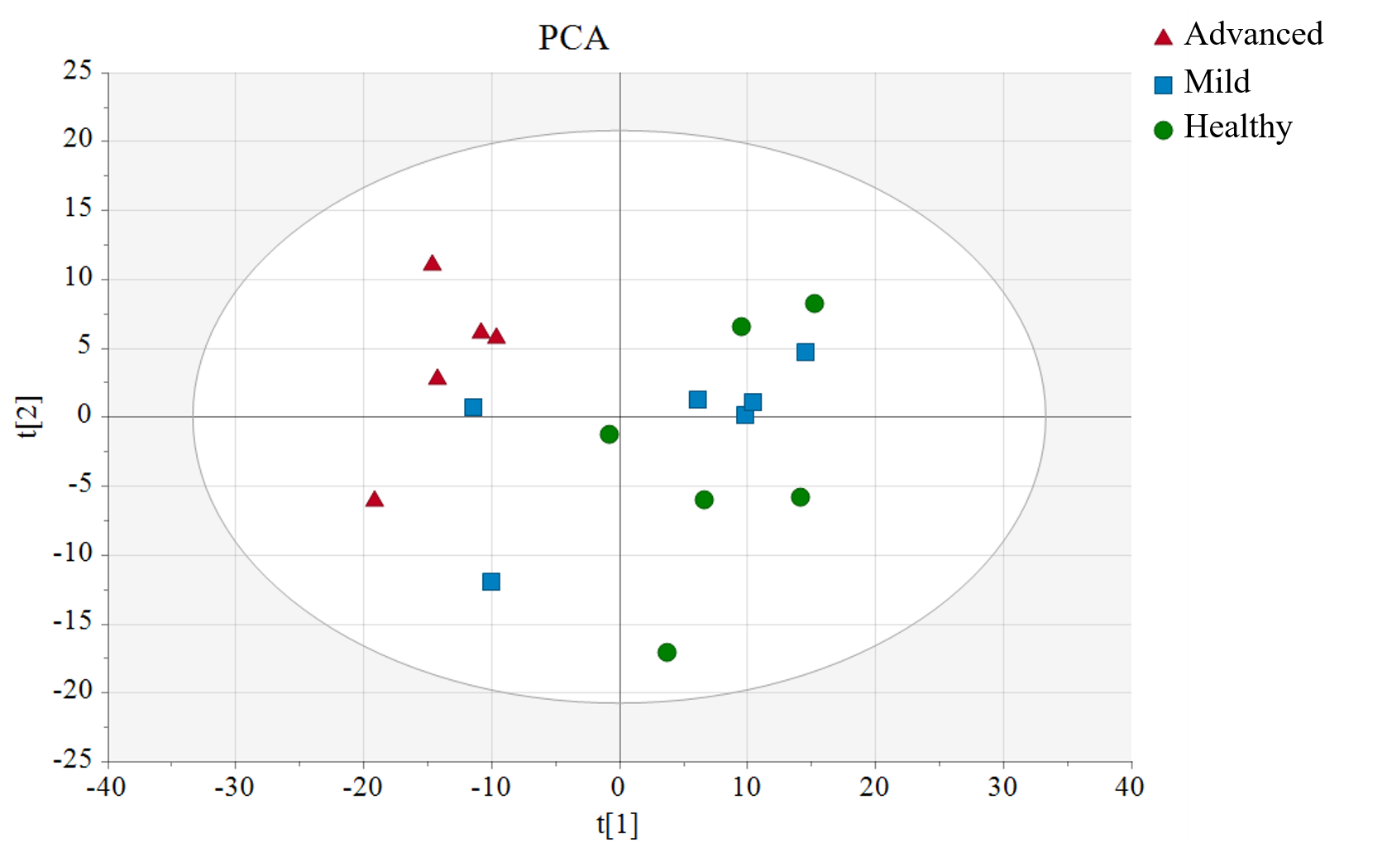
 **Figure S5:** Score plot PCA analysis for classes advanced, mild and healthy.
